# Role of Two-Dimensional Speckle-Tracking Echocardiography in Early Detection of Left Ventricular Dysfunction in Dogs

**DOI:** 10.3390/ani11082361

**Published:** 2021-08-10

**Authors:** Lina Hamabe, Ahmed S. Mandour, Kazumi Shimada, Akiko Uemura, Zeki Yilmaz, Kentaro Nagaoka, Ryou Tanaka

**Affiliations:** 1 Department of Veterinary Surgery, Faculty of Veterinary Medicine, Tokyo University of Agriculture and Technology, Tokyo 183-8509, Japan; dr_mandour@vet.suez.edu.eg (A.S.M.); ruiyue1221@gmail.com (K.S.); 2 Department of Animal Medicine (Internal Medicine), Faculty of Veterinary Medicine, Suez Canal University, Ismailia 41522, Egypt; 3 Department of Veterinary Surgery, Division of Veterinary Research, Obihiro University of Agriculture and Veterinary Medicine, Hokkaido 080-8555, Japan; anco@vet.ne.jp; 4 Department of Internal Medicine, Faculty of Veterinary Medicine, Uludag University, Bursa 16059, Turkey; zyilmaz@uludag.edu.tr; 5 Department of Veterinary Physiology, Faculty of Veterinary Medicine, Tokyo University of Agriculture and Technology, Tokyo 183-8509, Japan; nagaokak@cc.tuat.ac.jp

**Keywords:** echocardiography, speckle tracking echocardiography, strain, myocardial function, left ventricle, dog

## Abstract

**Simple Summary:**

Two-dimensional speckle-tracking echocardiography represents an advanced imaging technique that allows the analysis of global and regional myocardial function, cardiac rotation and synchronicity using deformation imaging. It has gained growing importance over the last decade, especially in human medicine as a method of evaluating myocardial function. This review aims to give an overview of the current understanding of this technique and its clinical applicability in the field of veterinary medicine with a focus on early detection of left ventricular dysfunction in dogs.

**Abstract:**

Two-dimensional speckle-tracking echocardiography (2D–STE) is an advanced echocardiographic technique based on deformation imaging that allows comprehensive evaluation of the myocardial function. Clinical application of 2D–STE holds great potential for its ability to provide valuable information on both global and regional myocardial function and to quantify cardiac rotation and synchronicity, which are not readily possible with the conventional echocardiography. It has gained growing importance over the past decade, especially in human medicine, and its application includes assessment of myocardial function, detection of subclinical myocardial dysfunction and serving as a prognostic indicator. This review illustrates the fundamental concepts of deformation analysis and gives an overview of the current understanding and its clinical application of this technique in veterinary medicine, with a focus on early detection of left ventricular (LV) dysfunction in dogs.

## 1. Introduction

Standard echocardiography is a widely used non-invasive method for evaluating myocardial function. In veterinary medicine, left ventricular (LV) fractional shortening (FS) is the most commonly used echocardiographic measurement of systolic function, readily obtained from LV chamber dimensions [1,2]. However, FS does not reflect the true systolic function since it only assesses myocardial shortening in a radial direction at a pair of specific myocardial segments even though myocardial contraction occurs in multiple directions [2]. Additionally, FS is known to be influenced by loading conditions [2]. To overcome these shortcomings, deformation imaging was developed to provide a comprehensive analysis of myocardial function, and the most widely used technique in both human and veterinary medicine is the 2D–STE.

## 2. What Is Two-Dimensional Speckle-Tracking Echocardiography?

The 2D–STE technique is advanced echocardiography that assesses myocardial function by quantifying myocardial deformation. It is based on the formation of “speckles” (natural acoustic markers) generated by interactions between myocardial tissue and ultrasound beams in standard gray scale two-dimensional (2D) echocardiographic images [3,4]. Tracking these speckles from one frame to another enables the analysis of myocardial movement throughout the entire cardiac cycle and provides information on deformation, which are measured as strain and strain rate (SR) [3,4].

Strain (Lagrangian strain, ε) is a unitless measurement of deformation of the myocardium over time, expressed as percent change from its original dimension. It is calculated as

ε = *L* − *L*_0_/*L*_0_,

where *L* is the instantaneous length, and *L*_0_ is the initial length [4,5]. SR (ε’) is the temporal derivative of strain expressed as s^−1^, representing the rate of deformation [4,5]. The lengthening, thickening and clockwise rotation of the myocardium are expressed as positive deformation, whereas shortening, thinning and counterclockwise rotation are negative [3].

## 3. Advantages and Disadvantages of 2D–STE

### 3.1. Advantages

The basis of 2D–STE is the B-mode image and strain parameters are calculated from the 2D displacement of the myocardium along the myocardial wall, whereas tissue Doppler-derived strain parameters are dependent on the angle between the beam and the direction of myocardial movement [3,4,6]. Therefore, 2D–STE is considered angle independent, which allows evaluation along different spatial orientations [3,4]. Additionally, since the strain and SR are measured using 2D intra-tissue velocities, these deformation parameters are also independent of cardiac translational movement and tethering, and this permits the differentiation between an active myocardial deformation and a passive displacement of the myocardium [3,4]. 

Furthermore, 2D–STE allows the simultaneous evaluation of global and regional myocardial function [4]. A six-segment model is the most frequently used, where the LV myocardium is divided into six segments, and regional strain and SR are the average values within each segment [5]. Not only does segmental analysis allow the identification of regional myocardial abnormalities, but it also allows the quantification of LV synchronicity, which is identified by the variability among each segment [7]. Global strain and SR are the average values of all segments, which represent the overall myocardial function [4,5].

The analysis of 2D–STE is performed using offline software, and its measurement involves the manual or semi-automatic tracing of the myocardial border by the operator. Then the software algorithm automatically tracks the speckles throughout the cardiac cycle. This semi-automatic method results in lower inter- and intra-operator variability of 2D–STE analysis [4].

### 3.2. Disadvantages

There are several factors to be wary of when using 2D–STE to analyze myocardial function. One is the potential measurement variability produced by software differences in the analytical algorithms used by different vendors [5,8]. Farsalinos et al. compared the strain measurements of 62 human volunteers having variable LV function obtained from nine different vendors, and they found moderate, but statistically significant, vendor variability with a maximum absolute differences up to 3.7% (*p* < 0.001) [8]. It was concluded that these should not have a major impact on clinical interpretation; however, ideally, the same echocardiographic machine and software should be used for subsequent examinations [8]. Additionally, offline analysis requires high-quality image data obtained at a high frame rate (optimally, 50–70 frames per second), and it is time consuming compared to conventional echocardiography, making 2D–STE disadvantageous for routine clinical use [4,6].

Lastly, although to a lesser degree than FS, studies of both humans and dogs have shown that strain and SR are also partially dependent on loading conditions; therefore, care should be taken when interpreting the measurements of deformation in disorders involving altered loading conditions [6,9,10]. It should be noted that parameters of deformation analysis are not measurements of contraction but rather an estimation of systolic function [6]. 

## 4. LV Deformation and 2D–STE 

**Figure 1:**
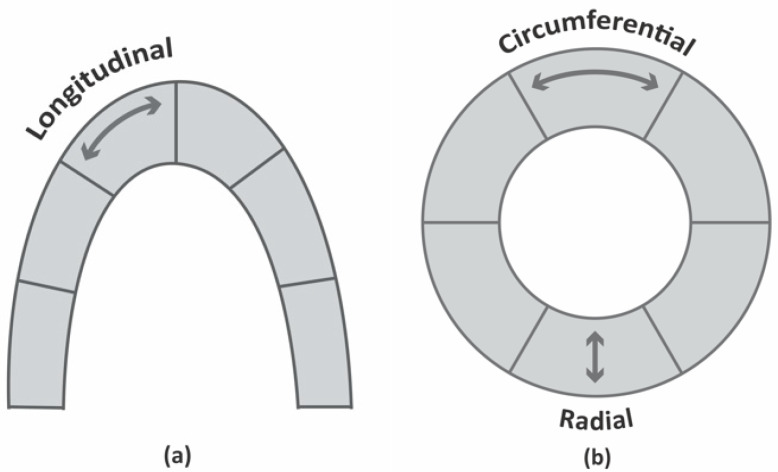
Schematic representation of LV deformation in three spatial orientations (**a**) longitudinal and (**b**) radial and circumferential. The longitudinal and circumferential strains show systolic shortening and diastolic stretching, while the radial strain shows systolic thickening and diastolic thinning.

**Figure 2:**
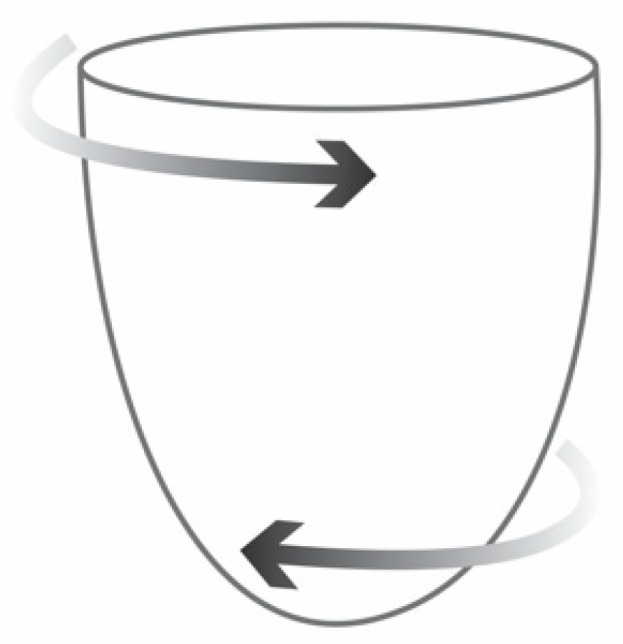
Schematic representation of LV rotation. The apex moves in counterclockwise direction and the base in clockwise direction during systole and then in opposite directions during diastole. The combination of two opposing movements results in twisting of the heart during systole and untwisting during early diastole along the long axis.

During cardiac contraction, LV deformation occurs in three spatial orientations: systolic shortening and diastolic stretching in the longitudinal and circumferential planes, and systolic thickening and diastolic thinning in the radial plane (Figure 1) [4]. Additionally, in systole, there is a counterclockwise movement of the apex and a clockwise movement of the base; in diastole, the direction is the opposite. This is known as rotation (Figure 2) [4,11].

### 4.1. Longitudinal Deformation

**Figure 3:**
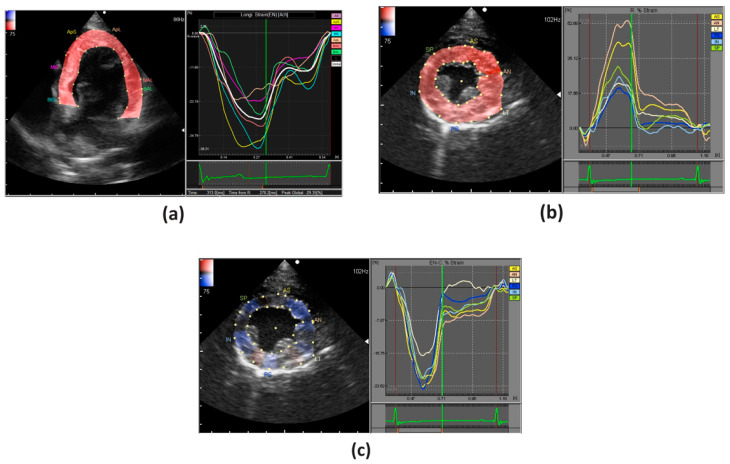
An example of strain analysis of the LV in a healthy dog; (**a**) longitudinal strain (apical 4-chamber view); (**b**) radial and (**c**) circumferential strains (parasternal short axis view). In longitudinal and circumferential strains, the deformation is illustrated by a negative curve during systole, whereas in radial strains it is illustrated by a positive curve.

Longitudinal strain is evaluated in apical 2-, 3-, 4-chamber views, and it represents myocardial deformation along the longitudinal axis (Figure 3a). In humans, it has been shown that global longitudinal strain measurements are superior, if not comparable, to the LV ejection fraction (EF) for inter- and intra-observer variability [8]. Additionally, polar projection of the longitudinal strains, the so-called bull’s-eye map, obtained from superimposing apical 2-, 3-, 4-chamber views, allows evaluation of regional myocardial changes and is useful in visualizing regional homogeneity [12].

Longitudinal fibers of the myocardium consist mainly of sub-endocardial fibers and most significantly contribute to the longitudinal myocardial function [13,14,15]. This has been demonstrated in both humans and dogs as these sub-endocardial fibers are shown to be most susceptible to ischemic changes since they are located the furthest from the epicardial coronary blood supply [14,15]. In humans, sub-endocardial changes to the myocardium are seen in ischemic injuries and in the first stages of various diseases such as arterial hypertension [4]. Therefore, longitudinal strain is considered to be a sensitive indicator of LV dysfunction and suitable for the early detection of sub-endocardial change. 

### 4.2. Radial & Circumferential Deformation 

**Figure 4:**
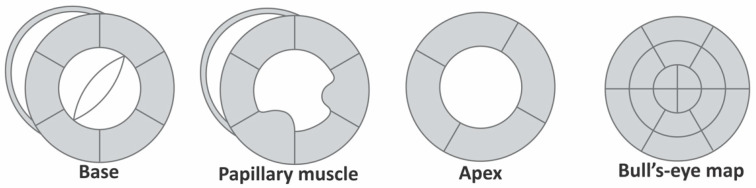
Schematic representation of short axis views at the base, papillary muscle and apical levels and bull’s-eye map created by superimposing all three.

Radial and circumferential strains are evaluated in parasternal short axis views at the level of basal, papillary muscles and apex (Figure 3b,c). Like the longitudinal deformation, a bull’s-eye map can be created by superimposing the parasternal short axis views of the base, papillary muscle and apical levels (Figure 4).

Myocardial contractility occurs in radial and circumferential directions, and radial and circumferential strains have been shown to be sensitive indicators of myocardial contractility in dogs [16]. It must be noted that radial and circumferential strains also increase to compensate for longitudinal dysfunction. This has been documented both in humans and dogs, indicating that increase in contractility does not always suggest improvement of myocardial function [4,17].

### 4.3. Rotational Deformation

Combining the two opposing movements at the apex and the base results in the twisting of the heart during systole and untwisting during early diastole along the long axis [4,11]. The rotational deformation is measurable using two parameters, twist and torsion [5]. Twist, expressed in degrees, is the difference in the systolic rotation in apical and basal levels of the short axis view, whereas torsion, expressed in degrees/cm, is the value of twist normalized to the length of LV cavity, which is twist divided by the distance between the apex and base [4,8]. LV rotation is thought to be a sensitive indicator of altered LV function in humans [4]. However, the task force recommendations by the European Association of Cardiovascular Imaging (EACVI) and the American Society of Echocardiography (ASE) consider both parameters to be poorly defined and stated they should be used with caution [8]. The terms “twist” and “torsion” are often used interchangeably in the veterinary literature, in most cases referring to the LV twist [11,18,19,20].

### 4.4. Synchronicity

LV dyssynchrony, defined as an uncoordinated contraction of LV, has detrimental effects on regional myocardial perfusion, metabolism and electrophysiology, and leads to compromised global LV systolic function and energy insufficiency [21]. In humans, it is a known major contributor of heart failure (HF) and a powerful predictor of mortality in patients with HF [21]. Although evaluation of LV dyssynchrony is feasible in all three directions of strain analysis, radial strain allows the most accurate detection of LV dyssynchrony and is considered most useful for humans [4,7]. It must be noted that there is no standardized method for the analysis of LV dyssynchrony. There is a variety of parameters in both the human and veterinary literature. The two most common are the maximal time delay between peak systolic strain of the earliest and latest segments and the standard deviation of time-to-peak systolic strain across the segments [7,10,22,23,24,25]. 

## 5. 2D-STE in Veterinary Medicine

### 5.1. Validation of 2D–STE in Canine Models

Table 1 shows a list of studies that investigated 2D–STE using canine experimental models. Sonomicrometry, which allows the measurements of the distance between ultrasonic crystals implanted in the myocardium, is often used as a reference method to validate 2D–STE. In the canine myocardial ischemic model, created by the transient occlusion of the left anterior descending coronary artery, 2D–STE showed adequate sensitivity in detecting changes in myocardial function produced by the alteration of regional myocardial blood flow, which had good correlation with the sonomicrometry [11,26,27,28]. A study by Adachi et al. demonstrated that during acute coronary blood-flow occlusion, a reduction in radial strain was observed to a lesser degree compared to the longitudinal and circumferential strains; however, the differences were statistically insignificant [13]. This may be due to a greater standard deviation in the radial strain, which may have resulted in lower accuracy [13]. Moreover, Stendahl et al. showed a similar correlation between 2D–STE and sonomicrometry in both radial and circumferential strains (r = 0.56 and r = 0.55, *p* < 0.001, respectively); nevertheless, the circumferential strain had better correspondence with a smaller bias [27]. These results suggest that the strain values from longitudinal and circumferential directions may be more favorable. The validation of twist between 2D–STE and sonomicrometry also showed good agreement (r = 0.94, *p* < 0.001) [11]. 

The usability of 2D–STE in dogs with HF was evaluated using tachycardia-induced myocardial dysfunction model [22,25,29,30,31]. These studies showed significant changes in systolic parameters of both conventional echocardiography and strain analysis in dogs with HF, which reflected the myocardial changes caused by high-electrical pacing [22,25,31]. With the analysis of rotation, the twist decreased significantly with the development of HF and returned to normal upon recovery, which suggests that the twist is a good indicator of myocardial function [30,31]. 

Arita et al. revealed that EF and tissue Doppler-derived dyssynchrony parameters were able to detect significant changes only in dogs with HF (with and without dyssynchrony) but not in dogs with dyssynchrony without HF [22]. Moreover, the M-mode derived dyssynchrony parameter was only able to detect significant changes in dogs with HF with dyssynchrony [22]. In comparison, both the radial and circumferential strains were able to detect significant differences in all three groups [22]. These strains not only detected dyssynchrony in HF better than the tissue Doppler- and M-mode-derived dyssynchrony parameters, but they effectively detected dyssynchrony without HF. It should be noted that Hamabe et al. observed a significant dyssynchrony only in the radial direction, and Arita et al. detected dyssynchrony in both directions with similar accuracy but with less variability in the radial direction [22,25]. These results may suggest the better sensitivity of radial strain to detect dyssynchrony. 

**Table 1:** Studies of canine experimental models with two-dimensional speckle tracking echocardiography (2D–STE).

Study	Model	Objective	Parameter	Outcome
Myocardial ischemic model				
Adachi et al. [13]	LAD coronary artery occlusion (*n* = 13)	Investigate changes in parameters of 2D–STE	LS, RS, CS	Reduction in RS occurred later than LS and CS No significant differences in diagnostic accuracy
Amundsen et al. [26]	LAD coronary artery occlusion (*n* = 9)	Validate 2D–STE against SM	long-, short-axis strains	Good correlation between STE and SM Long-axis strain: r = 0.90, *p* < 0.001 Short-axis strain: r = 0.79, *p* < 0.001
Helle-Valle et al. [11]	LAD coronary artery occlusion (*n* = 13)	Validate 2D–STE against SM	Twist	Good correlation between STE and SM Twist: r = 0.94, *p* < 0.001
Pirat et al. [28]	LAD coronary artery occlusion (*n* = 7)	Validate 2D–STE against SM	LS, CS	Good correlation between STE and SM LS: r = 0.83, *p* < 0.001 CS: r = 0.88, *p* < 0.001
Stendahl et al. [27]	LAD coronary artery occlusion (*n* = 7)	Validate 2D–STE against SM	RS, CS	Moderate correlation between STE and SM RS: r = 0.56, *p* < 0.001 CS: r = 0.55, *p* < 0.001 CS showed better correspondence with smaller bias
Tachycardia-induced heart failure model				
Arita et al. [22]	Dyssynchrony without HF (D) (*n* = 12) Dyssynchrony with HF (DHF) (*n* = 9) HF with narrow QRS (HF) (*n* = 8)	Evaluate synchronicity using CE, TDI and STE	Synchronicity	EF: Significant difference in HF, DHF (*p* < 0.05) TDI: Significant difference in DHF (*p* < 0.05) M-mode: Significant difference in DHF (*p* < 0.05) RS, CS: Significant difference in all D, DHF, HF (*p* < 0.05)
Hamabe et al. [25]	RV pacing at 250 bpm for 3 weeks (*n* = 5)	Compare CE with STE	RS, CS, synchronicity	Significant reduction of RS and CS (*p* < 0.01) Dyssynchrony only observed in radial direction (*p* < 0.05)
Kusunose et al. [30]	RV pacing at 220 bpm for 4 weeks (*n* = 7)	Establish normal values of STE Quantify impact of tachycardia-induced cardiomyopathy	LS, RS, CS, twist	Decrease in LS, RS, CS (*p* < 0.001) and twist (*p* < 0.05) Most profound effect in the LV apex (*p* < 0.001) RS had the largest relative decrease (*p* < 0.05)
Wong et al. [31]	RV pacing at 230–250 bmp for 2–4 weeks (*n* = 6)	Evaluate the effect of pacing using STE	LS, CS, twist, torsion	LS, CS, twist and torsion significantly decreased with HF and improved with recovery (*p* < 0.05)
Dyssynchrony model				
Mochizuki et al. [24]	LBB ablation (*n* = 10)	Determine diagnostic value of dyssynchrony parameters of STE	Synchronicity	Dyssynchrony parameters showed significant increase after ablation RS allowed detection of dyssynchrony with high sensitivity and specificity

bpm, beats per minute; CE, conventional echocardiography; CS, circumferential strain; EF, ejection fraction; HF, heart failure; LAD, left anterior descending; LBB, left bundle branch; LS, longitudinal strain; LV, left ventricle; RS, radial strain; RV, right ventricle; SM, sonomicrometry; TDI, tissue-Doppler imaging.

### 5.2. 2D-STE in Clinically Healthy Dogs

The normal values of strain analysis in dogs are comparable those of healthy humans (Table 2) [32,33]. Normal radial strain values show greater variability compared to longitudinal and circumferential strains, and the source of this variability is thought to be technical rather than biological [32]. The 2D–STE values in dogs tend to increase from base to apex with the highest value at the apex, which is also seen in healthy humans where alteration in this base-to-apex gradient of LV deformation is associated with various cardiac pathologies [20,30,34,35,36]. 

Chetboul et al. was the first to provide data on strain analysis in awake dogs [10]. Radial strain and SR of 37 clinically healthy dogs revealed adequate intra-observer repeatability (within-day variability) and reproducibility (between-day variability) with a coefficient of variation (CV) of less than 10% [10]. Likewise, a longitudinal strain had a CV of intra-observer repeatability and reproducibility and of inter-observer repeatability that was less than 10%, with the exception of inter-observer repeatability of LV free wall, which was 15.1% [37]. 

No correlation was found between age and systolic parameters of 2D–STE; however, between young and old dogs there were significant differences in diastolic deformations (early and late diastolic RS) that are consistent with increased ventricular stiffness and delayed relaxation resulting in reduced diastolic function, which is commonly observed with age [10,19]. Conflicting results in heart rate (HR) were reported, Chetboul et al. described a positive correlation with radial strain and SR (r = 0.41, *p* = 0.01; r = 0.56, *p* < 0.001, respectively), whereas, a study with controlled HR using right-atrial pacing showed no significant changes with increased HR [10,38]. Additionally, no correlation was found between body weight (BW) and strain parameters [10]. 

Twist had a reasonable intra-observer variability (within-day CV of 16.37% and between-day CV of 6.84%) and appeared not to be affected by HR or BW [18]. In healthy humans, twist was shown to increase with age, but studies in normal dogs revealed no correlation with age, which could be attributed to small sample sizes [18,19,20,39]. The 2D–STE analysis in dogs showed LV synchrony, and the synchrony parameters appeared to be independent of age, HR and BW [10,23].

**Table 2:** Normal values of two-dimensional speckle tracking echocardiography (2D–STE) in clinically healthy dogs.

Study	Subject	LS (%)	RS (%)	CS (%)	Twist (°)	Synchronicity (ms)	FS (%)
Chetboul et al. [10]	MB *n* = 37	-	46.7 ± 12.2 (26.1−69.2)	-	-	R STI: 15 ± 15 (0−49)	38.4 ± 6.0 (30.0−49.0)
Chetbou et al. [18]	MB *n* = 35	-	-	-	8.4 ± 3.8 (2.5−18)	-	8.4 ± 3.8 (2.5−18)
Griffiths et al. [23]	MB *n* = 10	-	-	-	-	R STI apex: 41.8 ± 17.9 base: 36.2 ± 19.0 R Ts_SD_ apex: 17.7. ± 7.2 base: 13.8 ± 7.3	-
Hamabe et al. [25]	Beagles *n* = 5	-	31.96 ± 7.12	−15.44 ± 1.5	-	R STI: 44.25 ± 17.6 C STI: 41.63 ± 12.68	34.7 ± 4.6
Kusunose et al. [30]	Mongrels *n* = 25	−18 ± 4	39 ± 20	−17 ± 4	8.1 ± 4.4	-	EF: 61 ± 8
Pedro et al. [34]	Great Danes *n* = 39	-	47.18 ± 12.00	−16.73 ± 2.58	-	-	27.8 ± 5.5
Smith et al. [40]	MB *n* = 20	-	43.9 ± 8.54	−20.9 ± 3.15	-	-	40 ± 6.16
Suzuki et al. [19]	Beagles Young (*n* = 17) Old (*n* = 15)	−14.8 ± 3.1 (−8.9 to −21.9) −14.9 ± 4.7 (−4.3 to −23.2)	52.4 ± 11.1 (27.7−70.0) 50.1 ± 12.3 (21.4−64.1)	−19.4 ± 4.4 (−10.1 to −25.8) −17.6 ± 2.5 (−13.3 to −21.2)	14.7 ± 4.6 (7.4−28.0) 13.6 ± 5.8 (3.9−25.2)	-	37.4 ± 7.3 (27.6−58.8) 37.6 ± 7.0 (25.4−48.7)
Suzuki et al. [17]	MB *n* = 20	−19 (−23 to −14)	55 (47−60)	−23 (−27 to −20)	-	-	39 (35−41)
Wess et al. [37]	MB *n* = 100	IVS: −16.89 ± 4.26 LVFW: −15.18 ± 5.86	-	-	-	-	-
Westrup et al. [20]	Irish wolfhounds *n* = 46	−16.2 ± 3.0 (−22.2 to −10.2)	Apical: 45.1 ± 10.4 (24.3−65.9) Basal: 36.9 ± 14.7 (7.5−66.3)	Apical: −24.8 ± 6.2 (−12.8 to −38.5) Basal: −15.9 ± 3.2 (−22.3 to −9.5)	11.5 ± 5.1 (1.3−21.7)	-	-

Data are expressed as mean ± standard deviations (range). C, circumferential; CS, circumferential strain; EF, ejection fraction; FS, fractional shortening; IVS, interventricular septum; LS, longitudinal strain; LVFW, left ventricular free wall; MB, mixed breed; R, radial; RS, radial strain; STI, synchrony time index; Ts_SD_, standard deviation of time to peak systolic segmental motion.

## 6. Clinical Application of 2D–STE 

### 6.1. Cardiac Disorders

#### 6.1.1. Myxomatous Mitral Valve Disease (MMVD)

MMVD is the most common acquired cardiac disease in dogs, characterized by chronic myxomatous degeneration of the mitral valve resulting in valvular dysfunction with secondary mitral valve regurgitation (MR) [41,42]. Although, LV systolic dysfunction is an important prognostic indicator, altered hemodynamic loading conditions in MMVD make using conventional echocardiography to assess LV function a challenge [42,43].

Smith et al. evaluated asymptomatic dogs with Stage B2 MMVD using 2D–STE (Table 3) [40,44]. When compared to the control group, the Stage B2 group had a significantly higher HR, greater LV size and LV systolic function, including both radial and circumferential strains, but LV dysfunction could not be identified [40]. Zois et al. observed similar results in increased strains, SRs and twist for dogs with congestive heart failure (CHF) compared to dogs with minimal or no MR (Table 3) [45,46]. These parameters increased with the severity of MMVD, which suggest augmented LV function [45,46]. However, longitudinal and radial strains and longitudinal SR showed curvilinear relationships with the left atrial-to-aortic ratio (LA/Ao), illustrating a decrease in LV function in dogs with CHF and severe left atrial enlargement [45]. These studies were unable to demonstrate LV dysfunction prior to the onset of clinical signs of CHF, and the hyperdynamic values of strain analysis may reflect a compensatory mechanism important for the preservation of LV function [46].

#### 6.1.2. Dilated Cardiomyopathy (DCM) 

DCM is the most common myocardial disease in dogs, characterized by progressive chamber dilation and impaired myocardial contractility [52,53,54,55]. It is well known that the asymptomatic “preclinical” DCM phase extends up to several years before any symptoms appear, during which the diagnosis can be challenging [52,53,54]. A study with 50 Great Danes diagnosed as preclinical DCM revealed an overall decrease in radial and circumferential strains and SRs at the base, papillary muscle and apical levels, with the greatest difference observed at the papillary muscles (Table 3) [34]. Additionally, a similar base-to-apex gradient of 2D–STE values was observed, but it was reduced in comparison with the clinically normal Great Danes, suggesting reduced systolic function in dogs with preclinical DCM [34]. 

Ro et al. reported serial changes of N-terminal pro-brain natriuretic peptide (NT–proBNP) and 2D–STE with disease progression observed in a Golden Retriever diagnosed with DCM and sub-aortic stenosis (SAS) (Table 3) [16]. Concomitant SAS makes this an atypical DCM; however, the dog presented with mainly DCM rather than SAS characteristics, and it fulfilled all proposed criteria for diagnosis of canine DCM [16]. It was speculated that the concomitant SAS resulted in worsened myocardial dysfunction and cardiac remodeling than DCM alone and resulted in a much shorter survival time [16]. Improvement in clinical signs, HR, NT–proBNP level and echocardiographic parameters of LV contractility were observed with treatment; however, segmental dyskinesia in the apical segment was detected with a regional analysis of 2D–STE. This regional deterioration of myocardial function, despite the increased contractility, suggested that the overall improvement of myocardial function did not necessary reflect the improvement of all myocardial segments. It also demonstrated that the regional analysis of 2D–STE was able to detect segmental myocardial dysfunction which was undetectable with NT–proBNP or conventional echocardiography [16]. They also revealed that the longitudinal strain and SR were the most sensitive and accurate indicators of the myocardial damage detected by NT–proBNP [16]. On the other hand, radial and circumferential strains and SRs were the most sensitive indicators of myocardial contractility, and these parameters also increased as compensation for longitudinal dysfunction, which was consistent with the findings of others [14,16,17]. Increased contractility does not always equate to improved myocardial function; therefore, it is necessary to evaluate all three directions of 2D–STE for a precise assessment of myocardial function [16]. 

#### 6.1.3. Patent Ductus Arteriosus (PDA)

PDA is the most common congenital heart defect in dogs, resulting from the failure of the ductus arteriosus to close, a normal fetal structure that shunts blood from the pulmonary artery to the aorta by bypassing the nonfunctional lung and normally closes soon after birth [56]. After birth, the rise in systemic pressure and the drop in pulmonary artery pressure cause blood to flow through the PDA from the aorta to the pulmonary artery (left-to-right shunt), resulting in pulmonary over circulation and volume overload of the left atrium and LV [56]. A study by Spalla et al. compared dogs with PDA with healthy controls and found a significant increase in LV dimensions, indicating LV overload in the PDA groups (Table 3) [47]. An increase in preload increases contractility by the Frank–Starling low, but EF and FS were not different between the two groups, which suggests the possible underestimation of contractility in dogs with PDA [47]. On the other hand, the 2D–STE parameters of longitudinal, radial and circumferential directions showed significant differences between the two groups, possibly suggesting that 2D–STE is a more sensitive indicator of systolic function [47]. 

Subsequently, Spalla et al. evaluated changes associated with the surgical closure of the PDA (Table 3) [48]. Ductal closure resulted in significant decreases in conventional echocardiographic parameters of LV dimensions and contractility including EF and FS as a result of the decreased preload and increased afterload [48]. A similar result was observed by Hamabe et al., where diastolic dimensions and FS decreased significantly (Table 3) [9]. Additionally, the PDA closure resulted in a significant reduction in radial and circumferential strains and SRs, whereas longitudinal strain and SR did not show any change [9,48]. The decrease in preload resulted in reduced tension on the myocardial wall and the radius of the LV, leading to reduced contraction of the radial and circumferential fibers, which may explain the reduced radial and circumferential strains and SRs [48]. On the other hand, longitudinal strain and SR are sensitive to early changes in systolic function, so the lack of changes may suggest that the PDA closure was not associated with systolic dysfunction [15,48]. Decreased contractility associated with the PDA closure, observed as decreased FS and radial and circumferential strains was most likely due to sudden changes in loading conditions. Such results suggest that the radial and circumferential strain parameters may be indicators of myocardial contractility, but they are also at least partially influenced by the loading condition. 

### 6.2. Non-Cardiac Disorders

#### 6.2.1. Systemic Inflammatory Response Syndrome (SIRS) 

SIRS is a clinical syndrome of infectious or non-infectious origin causing secondary multiple-organ dysfunction or death due to the excess release of cytokines. Cardiac dysfunction in SIRS has been demonstrated by an increase in cardiac biomarkers, such as NT–proBNP, cardiac muscle troponin T (cTnT) and lactate in dogs [57,58]. Although FS and LA/Ao have been shown to correlate significantly with survival-to-discharge, the detection of systolic dysfunction in dogs with SIRS has not been possible with conventional echocardiographic parameters, such as FS and EF [49,57]. A study by Corda et al. demonstrated that the endocardial longitudinal strain was able to identify systolic impairment in mild to moderate stages of SIRS, but it was not detected by the conventional echocardiography (Table 3) [49]. The endocardial longitudinal strain was significantly reduced (*p* = 0.001), without affecting the epicardial longitudinal or the radial strains [49]. The sub-endocardial myocytes are located the furthest from the epicardial coronary artery, and therefore it is considered most vulnerable to ischemia [14,15]. Consequently, SIRS-associated microvascular alterations resulting in myocardial ischemia may contribute to reduced endocardial longitudinal strain [59]. 

#### 6.2.2. Hyperadrenocorticism (HAC) 

HAC is an endocrinological disorder characterized by the chronic elevation of glucocorticoid in the blood. Systemic hypertension, LV hypertrophy and myocardial fibrosis are commonly recognized cardiovascular changes in humans and dogs with HAC [60,61]. In veterinary medicine, LV hypertrophy is reported anywhere from 47.3 to 68% of dogs with HAC [50,60]. Myocardial fibrosis results in increased LV stiffness and impaired LV relaxation causing LV diastolic dysfunction, which can be observed with the conventional echocardiography [50,60,61]. A study by Chen et al. showed a significant decrease in global strains and global peak systolic and early diastolic SRs in longitudinal and circumferential directions, suggesting impaired systolic and diastolic function in dogs with HAC (Table 3) [50]. Similar findings were reported in humans, where increased myocardial fibrosis in HAC patients resulted in both LV systolic and diastolic dysfunction [61]. In the study by Chen et al., conventional echocardiographic parameters of LV systolic function failed to detect any significant differences between dogs with HAC and controls, whereas 2D–STE was able to reveal a decrease in systolic function [50]. Such findings suggest that dogs with HAC may have subclinical systolic dysfunction that may be undetectable with conventional echocardiography [50].

#### 6.2.3. Parvoviral Enteritis (PVE)

PVE, caused by the infection of canine parvovirus (CPV-2), is presented as acute hemorrhagic gastroenteritis with high fatality in young dogs [62,63]. CPV-2 is transmitted via the fecal-oral route, and targets cells with high mitotic activity for viral replication, such as the intestinal epithelium and lymphoid tissue [62,63]. Myocarditis may also occur if the infection occurs within the first 2–3 weeks of life, when rapid myocardial cell proliferation takes place [62,63]. Moreover, myocarditis may also occur secondary to PVE, resulting from sepsis caused by the disruption of gastrointestinal barrier [64]. The hypovolemic state of the PVE caused by dehydration results in reduced preload, making detection of impaired myocardial function with conventional echocardiography difficult [51]. A study by de Abreu et al. demonstrated the presence of systolic dysfunction in dogs with PVE, indicated by impaired strains and SRs, while the conventional echocardiographic parameters failed to detect any changes (Table 3) [51]. Impairment of the longitudinal strain and SR at both endocardial and epicardial levels and circumferential strain and SR at the endocardium were observed in all dogs with PVE [51]. On the other hand, impairment of the circumferential strain in the epicardium was observed only in dogs that died from PVE, while circumferential SR in the epicardium remained normal [51]. Furthermore, regional analysis revealed the lowest circumferential strain and SR in the mid-septal epicardial segment in non-surviving dogs [51]. In fact, circumferential SR in that particular segment of less than 0.95 s^−1^ made possible the distinction between dogs with severe PVE and non-surviving dogs with 100% sensitivity and specificity [51]. Such results suggest that longitudinal strain and SR allow early detection of myocarditis in PVE, and a circumferential SR below 0.95 s^−1^ at the mid-septal epicardial segment may indicate a grave prognosis [51]. 

## 7. The Future of 2D–STE

In human medicine, the importance of 2D–STE analysis in evaluating myocardial function has been recognized, and a consensus document has been published by the EACVI and ASE to standardize deformation analysis [5]. Moreover, improvement in technology has allowed the development of 3D analysis. Similarly in veterinary medicine, the 2D–STE analysis has attracted great interest, expanding outside the simple analysis of LV and assessment of myocardial function. For example, several studies described the utility of 2D–STE for studying right-ventricular, right-atrial, and left-atrial function in dogs [65,66,67,68,69,70,71]. Additionally, studies demonstrated how 2D–STE analysis can be used to predict survival in MMVD and the onset of atrial fibrillation [72,73]. 

## 8. Conclusions

2D–STE analysis has gained increasing significance in human medicine over the past decade, and it is also becoming a promising tool for evaluating myocardial function in veterinary medicine. There is growing evidence that superior deformation parameters can serve as an indicator of myocardial function compared to conventional parameters for early diagnosis of myocardial dysfunction and as a possible prognostic indicator in both human and veterinary medicine. Additional information provided by the analysis of rotation, synchronicity and bull’s-eye maps by the 2D–STE analysis allow more comprehensive and accurate evaluation of myocardial function. There is no doubt that further development will allow deformation analysis to mature and play an important role in routine clinical use, with better diagnostic accuracy and reliability for dogs.

## Figures and Tables

**Table 3:** Clinical studies evaluating LV myocardial function in various disorders in dogs using two-dimensional speckle tracking echocardiography (2D–STE).

Study	Disorder	Parameter	Outcome
Myxomatous mitral valve disease (MMVD)			
Smith et al. [40]	Dogs with MMVD Stage B2 (*n* = 20)	RS, RSR, CS	Dogs with MMVD had significantly higher HR, LV size and LV systolic function, including CS, RS, RSRs
Zois et al. [45]	Dogs with various stages of MMVD (*n* = 93)	LS, LSR, RS, RSR, synchronicity	Increase in LS, LSR, RSR were observe with increasing severity of MMVD Curvilinear relationships with LA/Ao with decrease in deformation parameters pass LA/Ao ratio of 2.1 were seen with LS, LSR, RS, RSR
Zois et al. [46]	Dogs with various stages of MMVD (*n* = 97)	CS, CSR, twist	Dogs with CHF had increased CS, CSR, twist compared to dogs with mild MMVD CS increased with MR severity, and CSR and twist decreased with increased LVIDs
Dilated cardiomyopathy (DCM)			
Pedro et al. [34]	Great Dane with pre-clinical DCM (*n* = 50)	RS, RSR, CS, CSR at apical, PM and base levels	Decrease in RS, RSR, CS, CSR with greatest difference observed at PM Similar base to apex gradient was observed but reduced in comparison to the normal control
Ro et al. [16]	Golden Retriever with Subaortic stenosis and DCM (*n* = 1)	LS, LSR, RS, RSR, CS, CSR, bull’s-eye map	LS and LSR showed good correlation with myocardial damage detected by NT–proBNP RS, RSR, CS, CSR showed good correlation with of myocardial contractility Bull’s-eye map revealed regional deterioration of myocardial function despite increased contractility
Patent Ductus arteriosus (PDA)			
Hamabe et al. [9]	Dogs with PDA (*n* = 17)	RS, RSR, CS, CSR	Closure of PDA resulted in significant reduction of both FS and 2D–STE parameters
Spalla et al. [47]	Dogs with PDA (*n* = 34)	LS, LSR, RS, RSR, CS, CSR	Increased CE parameters of LV dimensions and 2D–STE parameters in dogs with PDA, whereas EF and FS did not differ
Spalla et al. [48]	Dogs with PDA (*n* = 25)	LS, LSR, RS, RSR, CS, CSR	Statistically significant decreases in all CE parameters were observed RS, RSR, CS, CSR decreased, whereas LS and LSR did not change
Systemic inflammatory response syndrome (SIRS)			
Corda et al. [49]	Dogs with SIRS (*n* = 17)	Endocardial and epicardial LS, LSR, RS, RSR	Endocardial LS was able to identify systolic impairment in dogs with SIRS, but not by the CE Endocardial LS was significantly reduced without affecting epicardial LS or RS
Hyperadrenocorticism (HAC)			
Chen et al. [50]	Dogs with PDH (*n* = 10) Dogs with ADH (*n* = 9)	LS, LSR, CS, CSR	Significant decrease in LS, LSRs, LSRe, CS, CSRs, CSRe CE revealed no significant changes in LV systolic function
Parvoviral enteritis (PVE)			
De Abreu et al. [51]	Dogs with mild PVE (*n* = 15) Dogs with severe PVE (*n* = 13) Dogs dead from PVE (*n* = 9)	Endocardial and epicardial LS, LSR, CS, CSR, RS, RSR	Strains and SRs were significantly reduced in all dogs, whereas CE parameters did not change Endo- and epicardial LS and LSR and endocardial CS and CSR were impaired in all dogs with PVE Epicardial CS was impaired only in dogs died from PVE, while epicardial CSR remained unchanged CSR of less than 0.95 s^−1^ allowed distinction between dogs with severe PVE and non-surviving dogs with 100% sensitivity and specificity

ADH, Adrenal-dependent hyperadrenocorticism; CE, conventional echocardiography; CS, circumferential strain; CSR, circumferential strain rate; CSRe, circumferential strain rate in late diastole; CSRs, circumferential strain rate in peak systole; EF, ejection fraction; FS, fractional shortening; HR, heart rate; LA/Ao, left atrium to aorta ratio; LS, longitudinal strain; LSR, longitudinal strain rate; LSRe, longitudinal strain rate in late diastole; LSRs, longitudinal strain rate in peak systole; LV, left ventricle; LVIDs, systolic LV internal diameter; NT–proBNP, N-terminal pro-brain natriuretic peptide; PM, papillary muscle; PDH, pituitary-dependent hyperadrenocorticism; PVE, parvoviral enteritis; RS, radial strain; RSR, radial strain rate.
